# Effect of High-Pressure Processing Treatment on the Physicochemical Properties and Volatile Flavor of *Mercenaria mercenaria* Meat

**DOI:** 10.3390/molecules29184466

**Published:** 2024-09-20

**Authors:** Xingli Xue, Di Wang, Min Li, Yongren Li, Yongjun Guo, Xiaoqing Ren, Chunsheng Li

**Affiliations:** 1 National R & D Branch Center for Conventional Freshwater Fish Processing (Tianjin), College of Food Science and Bioengineering, Tianjin Agricultural University, Tianjin 300392, China; 15249535767@163.com (X.X.); 13472377731@163.com (M.L.); 2 Key Laboratory of Aquatic Product Processing, Ministry of Agriculture and Rural Affairs of the People’s Republic of China, National Research and Development Center for Aquatic Product Processing, South China Sea Fisheries Research Institute, Chinese Academy of Fishery Sciences, Guangzhou 510300, China; wangdi1991624@hotmail.com; 3 College of Fisheries, Tianjin Agricultural University, Tianjin 300392, China; lyr1018@163.com (Y.L.); guoyongjun@tjau.edu.cn (Y.G.)

**Keywords:** high-pressure processing, *Mercenaria mercenaria*, quality, volatile flavor compounds

## Abstract

High-pressure processing (HPP) technology can significantly improve the texture and flavor of *Mercenaria mercenaria*. This study aimed to investigate the effect of HPP treatment with varying levels of pressure (100, 200, 300, 400, 500, and 600 MPa) and a holding time of 8 min at 20 °C on the physicochemical properties and volatile flavors of *M. mercenaria*. The significant changes in hardness, resilience, and water holding capacity occurred with increasing pressure (*p* < 0.05), resulting in improved meat quality. Scanning electron microscopy (SEM) was utilized to observe the decomposition of muscle fibers in *M. mercenaria* due to varying pressures, which explains the differences in texture of *M. mercenaria*. Different pressure treatments also had an influence on the volatile flavor of *M. mercenaria*, and the quantities of low-molecular-weight aldehydes (hexanal, heptanal, and nonanal) with a fishy taste decreased dramatically following 400 and 500 MPa HPP treatments. Furthermore, the level of 2-Methylbutyraldehyde, which is related to sweetness, increased significantly following 400 MPa HPP treatment. The study found that 400 MPa HPP treatment resulted in minor nutrient losses and enhanced sensory quality. The results of this study provide a theoretical basis for the application of HPP treatment to *M. mercenaria*.

## 1. Introduction

*Mercenaria mercenaria*, commonly known as the north quahog, belongs to the genus of *Mercenaria*. Native to the east coast of the United States and Canada [1], this species exhibits rapid growth, strong environmental adaptability, and resistance to varying temperatures and salinity levels [2]. Since its introduction to China, the aquaculture area dedicated to *M. mercenaria* has expanded to nearly 100,000 hectares, resulting in a significant annual production value in the billions of dollars. *M. mercenaria* is also popular with consumers due to its delicious flavor and high nutritional value, with nutrients such as omega-3 fatty acids, iron, zinc, vitamin A, and vitamin B12 [3,4]. Currently, traditional thermal processing methods to deal with *M. mercenaria* have limitations; the product temperature rises during the heat transfer process, causing changes in product quality, such as the texture being hardened, as well as nutrient content and flavor being lost. The use of HPP eliminates the problem of traditional thermal processing [5,6].

High-pressure processing (HPP) technology is a non-thermal method increasingly embraced by the food industry. It entails using a liquid as a pressure-transmitting medium to subject food to pressures ranging from 100 to 1000 MPa [7]. It can deactivate pathogenic and spoilage microorganisms and preserve the original nutrients in food [8]. HPP technique has been shown to effectively reduce initial microbial counts in aquatic products and inhibit microbial growth during storage, thus delaying spoilage and extending shelf life. Erkan et al. [9] have demonstrated that 330 MPa treatment for 5 min can extend the shelf life of *skipjack tuna* at 4 °C by an additional 3 days. Applying HPP to *Penaeus vannamei* results in a reduction of total bacterial counts by 0.37 logCFU/g and 1.29 logCFU/g at pressure levels of 270 MPa and 435 MPa, respectively [10]. Moreover, HPP treatment has been shown to improve the original nutritional value of aquatic products and enhance their flavor. Chen Z. et al. [11] have found that the 300 MPa HPP treatment combined with magnetic field storage can delay protein and lipid oxidation in grass carp fillets, inhibit the production of negative volatiles, and preserve fillet quality during storage. M.S. et al. [12] have demonstrated that pressure and holding times of HPP can influence the contents of free fatty acids and amino acids in clams.

However, despite all these studies, it is recognized that the impact of HPP technology on the sensory and nutritional qualities of *M. mercenaria* has received less attention and investigation, with prior studies focused only on the impacts of HPP technology on *M. mercenaria*’s microbiological safety [13]. Hence, the impact of HPP treatment on the physicochemical properties and sensory properties of *M. mercenaria* was studied in this study. The optimal conditions for HPP treatment of *M. mercenaria* from the point of the physical and chemical indicators were analyzed. The structure changes in *M. mercenaria* after HPP treatment were evaluated using scanning electron microscopy (SEM). Subsequently, the volatile compounds of *M. mercenaria* were analyzed and identified by GC-IMS to elucidate the effect of ultrahigh-pressure treatment on the flavor profile of *M. mercenaria*. These findings offer a theoretical foundation for using HPP with *M. mercenaria*.

## 2. Results and Discussion

### 2.1. Effect of HPP Treatment on Basic Nutrients

As shown in Table 1, the dry weight protein content of *M. mercenaria* in the untreated group was recorded at 66.92%. Conversely, the dry weight protein content in all experimental groups exhibited a notable decrease compared to that in the control group (*p* < 0.05), with no significant variance between the 400 MPa and 500 MPa treatment groups. The decrease in dry weight protein content is attributed to the loss of soluble proteins due to denaturation and aggregation of proteins caused by HPP. This finding is supported by the work of Oliveira F.A. et al. [14]. The initial dry weight fat content of *M. mercenaria* in the untreated group was measured at 7.21%, with a corresponding dry weight ash content of 21.32%. While the fat content displayed a decreasing tendency in dry base measurements, the difference was not statistically significant. This change in the lower fat content in *M. mercenaria* can be explained by the fat in the *M. mercenaria* dissolving and spilling as pressure increased [15].

In contrast, the ash content in dry base evaluations demonstrated a decreasing pattern; the decrease in ash content might be due to the release of inorganic salts from the body under pressure, leading to a lower ash content [14].

### 2.2. Effect of HPP Treatment on pH

**Figure 1:**
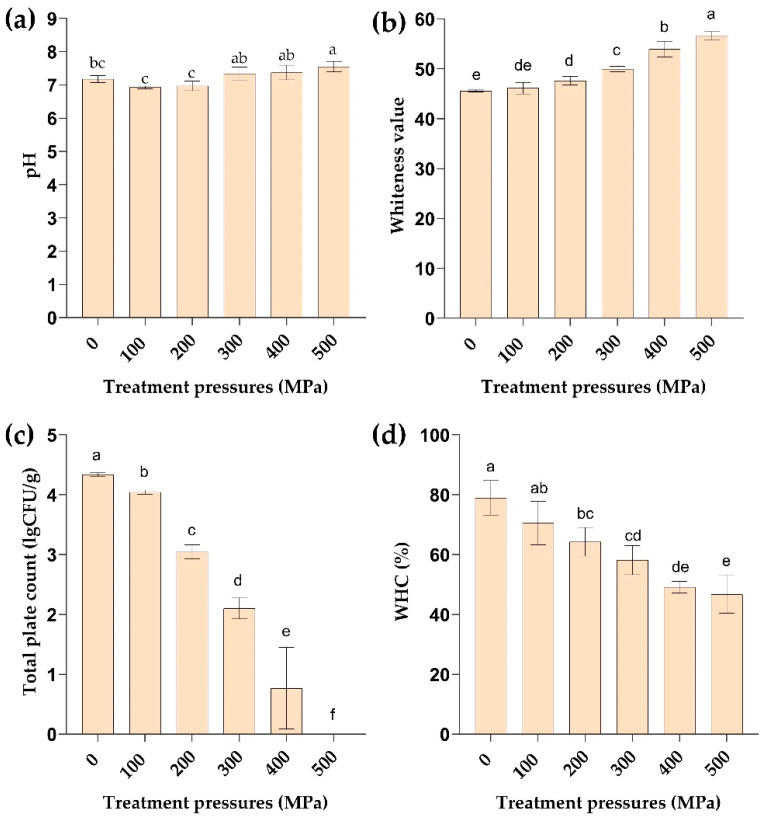
Effects of different pressures on (**a**) pH, (**b**) whiteness value, (**c**) total number of colonies, and (**d**) WHC of *M. mercenaria*. f indicates that the total number of colonies is less than 10 CFU/g. Different letters indicate significant differences at *p* < 0.05, and the same letter indicates a non-significant difference at *p* > 0.05.

After the death of aquatic products, the muscle tissues undergo glycolysis, resulting in the production of lactic acid and subsequent reduction in pH. Over time, microbial activity and enzymatic processes decompose the proteins in aquatic products into alkaline compounds such as ammonia and amines, leading to an increase in pH value [16]. Thus, pH serves as a significant indicator reflecting changes in the concentration of free hydrogen ions and hydroxide ions in the sample to assess the quality of aquatic products. The impact of different pressures on the pH levels of clams is shown in Figure 1a. At 100 MPa and 200 MPa, the pH levels slightly decreased compared with that of the control group, but the difference was not significant. This could be attributed to the relatively lower degree of protein denaturation at lower pressures, wherein the glycolysis process in the muscle tissue generates acidic byproducts like lactic acid and phosphocreatine, resulting in a decreased pH. The difference was significant between the 300–500 MPa treatment groups and the control group (*p* < 0.05); as the pressure continued to increase over 300 MPa, the pH levels obviously increased, especially at 500 MPa, displaying a 5.3% increase compared to the control group. This rise in pH can be ascribed to the extensive protein denaturation induced by high pressure, consequently leading to the protein decomposition into various alkaline substances. Oliveira F A et al. [15] have posited that the rise in pH in muscle tissues under HPP conditions is attributable to the expansion of muscle proteins, which exposes alkaline groups while burying acidic groups. Similarly, Sareevoravitkul et al. [17] have found that the high-pressure treatment results in an increase in the pH value of bluefish gel, attributed to the formation of nitrogen compounds, such as the deamination of free amino acids.

### 2.3. Effect of HPP Treatment on Color

The color of aquatic products is a crucial indicator of their sensory quality, playing a significant role in consumer acceptability. The impact of varying pressures on the whiteness of *M. mercenaria* is depicted in Figure 1b. As illustrated, the whiteness value of *M. mercenaria* exhibited an increasing trend with rising pressure levels. Notably, there was no notable distinction between the 100 MPa treatment group and the control group. However, a significant increase in whiteness value was observed after treatment over 200 MPa (*p* < 0.05), with the highest whiteness value recorded in the 500 MPa treatment group.

These findings suggest that the increase in whiteness value may be attributed to the denaturation of muscle fiber proteins and sarcoplasmic proteins after HPP treatment. This denaturation process alters the surface characteristics of the sample, impacting the ratio of light absorption and reflected light on the surface, consequently producing a whitening effect [18,19]. Additionally, Hughes et al. [20] have posited that pressure can cause a tighter muscle fiber structure within the meat, resulting in internal muscle fiber accumulation and sarcoplasmic refraction, thereby contributing to a whitening appearance.

### 2.4. Effect of HPP Treatment on the Total Number of Bacterial Colonies

The growth of spoilage microorganisms plays a crucial role in the deterioration of aquatic product quality. Therefore, the total number of bacteria serves as a key indicator for assessing the quality of aquatic products. As illustrated in Figure 1c, a significant decrease in the total number of colonies in *M. mercenaria* is observed with increasing pressure levels (*p* < 0.05). Specifically, the total number of colonies in the control group samples was 4.3 logCFU/g. Following HPP treatment at 300 MPa for 8 min, the total number of colonies decreased to 2.1 logCFU/g. Subsequently, after treatment with 400 MPa, the total number of colonies decreased to 0.8 logCFU/g. Finally, in the treatment with 500 MPa for 8 min, the total number of colonies decreased to non-detectable levels. The effectiveness of HPP treatment in reducing the total bacterial count has also been reported in other aquatic products [21,22,23], thereby prolonging their shelf life. This phenomenon is attributed to the disruption of cell membrane structures by HPP treatment, hindering the proliferation of spoilage bacteria and consequently eliminating microorganisms. These findings underscore the efficacy of HPP technology in diminishing microbial content to ensure product freshness and quality and minimize potential safety hazards for consumers.

### 2.5. Effect of HPP Treatment on Water Holding Capacity

Water holding capacity (WHC) is an important index to evaluate the quality of aquatic products. The strength of water holding directly affects consumers’ evaluation of the taste and texture of products [24,25]. The effect of HPP treatment on the water holding of *M. mercenaria* is shown in Figure 1d. With the increase in pressure, the WHC showed a downward trend. Compared with the control group, the decrease in WHC was not significant at 100 MPa but showed significantly over 200 MPa (*p* < 0.05). Xing-Sheng Ma et al. [26] have found that excessive HPP treatment leads to a decline in the water holding performance of surimi, which may be related to the fracture of the three-dimensional network structure in surimi gel. Lakshmanan et al. [27] have also found that HPP can lead to a reduction in the water holding capacity of freshly smoked fish, suggesting a correlation between water holding and protein interactions. This relationship is influenced by various factors, including pH and the specific structural characteristics of the muscle tissue in the sample.

### 2.6. Effect of HPP Treatment on Texture

**Figure 2:**
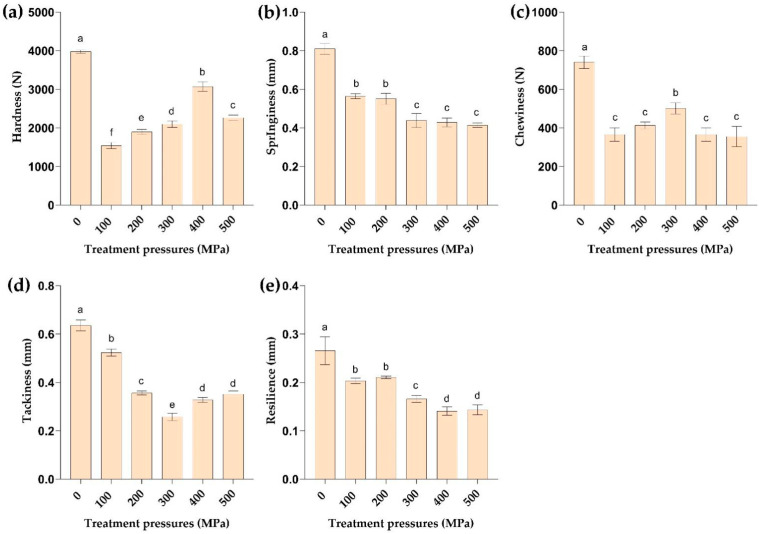
Effect of different pressures on the texture characteristics of *M. mercenaria*, including (**a**) hardness, (**b**) springiness, (**c**) chewiness, (**d**) tackiness, and (**e**) resilience. Different letters indicate significant differences at *p* < 0.05, and the same letter indicates a non-significant difference at *p* > 0.05.

The texture of meat determines the overall quality of the food and influences the assessment of product quality by consumers. Texture characteristics mainly include hardness, springiness, chewiness, tackiness, and resilience [28,29]. The *M. mercenaria* changes in texture characteristics may be related to protein denaturation-aggregation [25], actin–myosin interactions [30], α-actin release [31], and tissue compression [32]. As shown in Figure 2, the hardness of *M. mercenaria* increased and then decreased, with a significant drop after reaching 400 MPa compared to the control group (*p* < 0.05). The change in hardness could be attributed to the outflow of clam juice caused by the HPP treatment on the one hand, and the shortening of muscle segments caused by muscle tissue fiber compression on the other hand, followed by muscle fiber tissue rearrangement and structural changes, which resulted in the increase in hardness [33]. When the pressure reached 400 MPa, the protein molecules depolymerized and the protein secondary structure was disrupted, resulting in a reduction in hardness in the 500 MPa treatment group. Furthermore, the chewiness of *M. mercenaria* followed the hardness trend, and chewiness began to decline once the pressure reached 300 MPa, which was consistent with the study of Qiaoyu Liu [28].

Figure 2 additionally demonstrates that the springiness of *M. mercenaria* decreased with increasing pressure, and the change tended to level off when the pressure was greater than 300 MPa and was significantly lower than that of the control group, which was attributed to the hydrolysis of endogenous cathepsin and the degradation of myofibrillar proteins, which led to the decrease in elasticity. Furthermore, the tackiness of *M. mercenaria* decreased initially and then increased after the pressure reached 300 MPa, which could be attributed to myosin aggregation and denaturation in *M. mercenaria* products caused by HPP treatment [34]. Thus, HPP treatment can provoke changes in the interactions between protein molecules and lead to significant structural changes in myofibrils, with important effects on secondary, tertiary, and quaternary structures [35].

### 2.7. Effect of HPP Treatment on Tissue Structure

**Figure 3:**
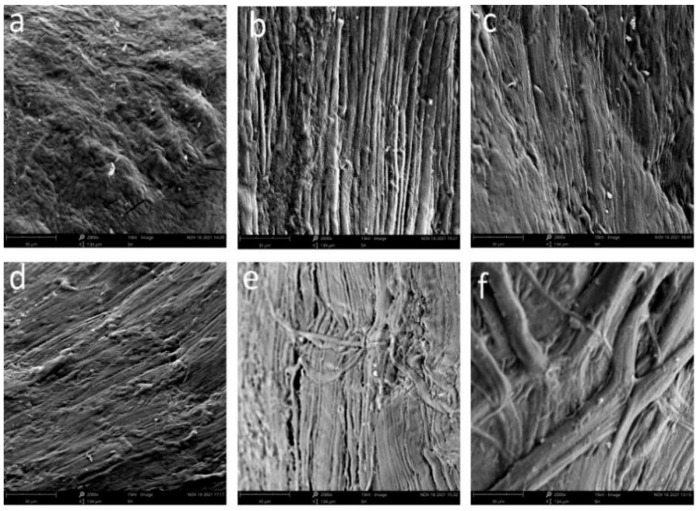
Tissue structure of clam meat using SEM (×2000) under (**a**) 0 Mpa, (**b**) 100 Mpa, (**c**) 200 Mpa, (**d**) 300 Mpa, (**e**) 400 Mpa, and (**f**) 500 Mpa.

The quality of aquatic products is affected by the density of muscle tissue structure. The influence of different pressures on the muscle structure of *M. mercenaria* is shown in Figure 3. The surface muscle structure of *M. mercenaria* meat treated with HPP was significantly different from that of the control sample. The myofibrillar tissue of the longitudinal muscle of fresh clam showed an uneven network structure. After 100 MPa HPP treatment, the muscle tissue contracted and deformed along the fiber axis and gathered to form a flat and compact structure. At 200 MPa, the arrangement of myofibril became compact. Under the condition of 300 MPa, the surface of muscle fibers showed a flocculent structure, the cohesion of myofibrillar fibers was the densest, and the gap between myofibrillar fibers was the smallest. At 400 MPa, the pore diameter between the myofibrils became loose, broken, or collapsed and basically maintained an orderly arrangement, while the myotome contraction was serious. As the pressure increased to 500 MPa, the muscle fiber structure on the muscle surface became coarse, and some fibers even broke, indicating that excessive pressure could also cause damage to muscle tissue [36,37]. Liu et al. [28] have shown that HPP can change the MP structure, which may be due to the degradation of the myofibrillar protein structure. A similar result has been found that HPP treatment has an impact on the muscle tissue structure of Atlantic salmon [38]. This study indicates that high pressure can lead to contraction of myogenic fibers and widening of the extracellular space (ECS); as pressure increases, connective tissue is destroyed and fibers cannot be held together.

### 2.8. Effect of HPP Treatment on Sensory Quality

**Figure 4:**
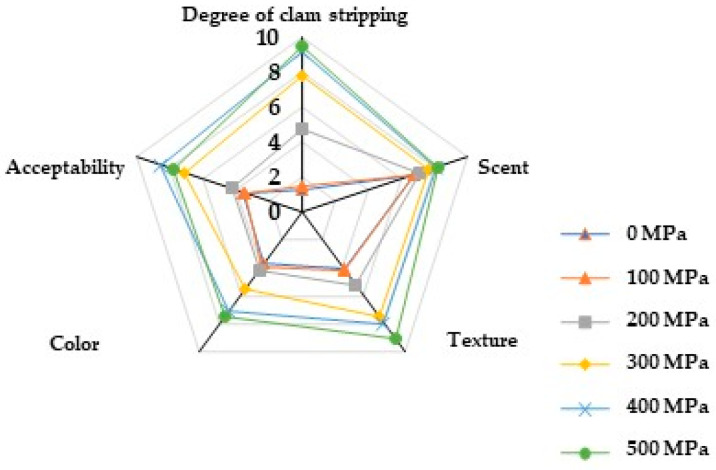
Effects of different pressures on sensory evaluation of *M. mercenaria*.

Sensory evaluation is a fundamental approach to assessing food quality. The impact of HPP treatment on the sensory attributes of *M. mercenaria* is outlined in Figure 4 and Table 2. With the increasing pressure levels, a visible separation of the closed shell muscle from the shell occurred, leading to an increase in gelatinization and fragility [39]. Regarding aroma, minimal distinctions were noted among the treatment groups, yet an enhancement in odor was observed in the high-pressure treatments of 400 MPa and 500 MPa when compared to the control group. The removal of some fishy volatile compounds and the generation of sweet aromatic compounds under 400 MPa and 500 MPa pressures rendered the odor more appealing to consumers. The color of *M. mercenaria* transitioned from yellowish to milky white after post-HPP treatment. Initially, the hardness and chewiness of the sample increased before declining with rising pressure levels, ultimately leading to a softer texture. The protein denaturation and aggregation resulted in the initial firmness of the sample tissues as pressure increased, followed by a subsequent decrease in hardness and chewiness upon complete protein disintegration. High acceptability scores were noted for the HPP treatments at 400 MPa and 500 MPa.

### 2.9. GC-IMS Spectrum Analysis

**Figure 5:**
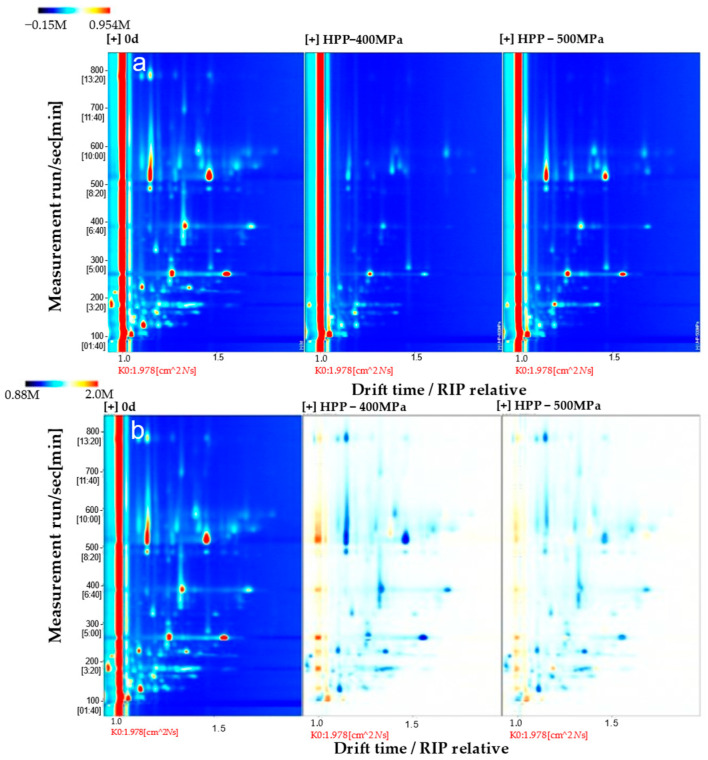
Qualitative analysis of GC-IMS spectra of *M. mercenaria* treated under different pressures before (**a**) and after (**b**) deducting control.

To explore the effect of HPP treatment on the flavor of clams, GC-IMS technology was used to analyze and identify the volatile flavor substances of clams treated with different pressures. The types and concentrations of volatile substances can be accurately compared in the 2D topographic map. There were obvious differences in the types and concentrations of volatile substances among different treatment groups. The difference comparison model was used to compare the differences between different samples. The spectrum of *M. mercenaria* without HPP treatment was selected as the reference, and the spectra of other samples were deducted. If the two volatile substances are consistent, the background after deduction is white, while red means that the concentration of the substance is higher than the reference, and blue means that the concentration of the substance is lower than the reference [40,41]. As shown in Figure 5, the types and contents of the VOCs in *M. mercenaria* in the control group were the highest, followed by those in the 500 MPa and 400 MPa treatment groups.

**Figure 6:**
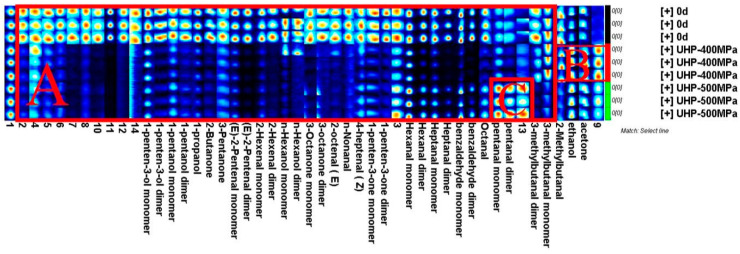
Fingerprint of volatile substances of *M. mercenaria* under different pressures.

To make a more intuitive and comprehensive comparison of the differences in volatile compounds among the samples of the three groups of *M. mercenaria*, the fingerprint of volatile substances was generated with the plug-in application Gallery Plot in Laboratry Ananlytical Viewer (LAV), the GC-IMS device’s built-in analysis software (2.2.1.) [42]. The fingerprint of the volatile substances of the three groups of *M. mercenaria* is shown in Figure 6. The substances on the left side of region A were the highest in the control group, and the concentrations of substances on the right side in the control group and 500 MPa group were consistently higher than that of the treatment group at 400 MPa. The main substances in region A were 1-penten-3-ol (monomer, dimer), 2-butanone, 3-pentanone, (E)-2-pentenal (monomer, dimer), 2-hexenal (monomer, dimer), n-hexanol (monomer, dimer), 3-octanone (monomer, dimer), (E)-2-octenal, (Z)-4-heptenal, 1-pentene-3-one (monomer, dimer), hexanal (monomer, dimer), heptanal (monomer, dimer), benzaldehyde (monomer, dimer), octanal, pentanal (monomer, dimer), and 3-methylbutanal (monomer, dimer). The substances in region B were the highest in the 400 MPa group, and the main substances were 2-methylbutanal, ethanol, and acetone, while the substances in region C were the highest in the 500 MPa treatment group, and pentanal (monomer and dimer) was the main substance.

**Figure 7:**
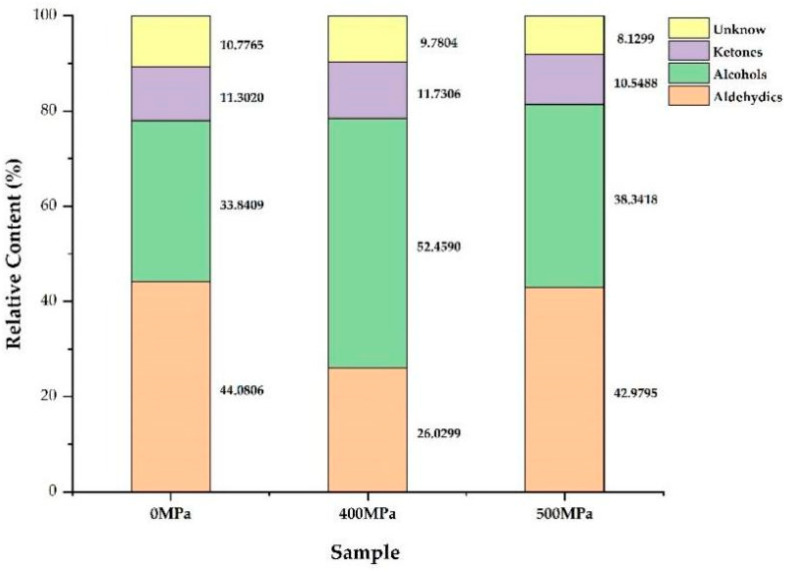
Percentage of volatile compounds in *M. mercenaria* under different HPP treatments.

By comparing the retention time and migration time of the characteristic volatile components, the retention index of each volatile component was calculated, and the qualitative analysis of volatile substances was performed by matching the NIST 2014 database and IMS database built in the GC-IMS application software. Table 3 and Figure S1 show that 48 compounds were identified, including 19 aldehydes, eight alcohols, seven ketones, and 14 unknown compounds. A comparison of VOCs is shown in Figure 7. According to statistical analysis, *M. mercenaria* treated with 400 MPa and 500 MPa HPP and the control group had comparable VOC levels. However, the VOC of the three groups of *M. mercenaria* differed significantly (*p* < 0.05) in peak intensity. Specifically, the aldehyde content decreased and the alcohol content increased in *M. mercenaria* after HPP treatment. In addition, the content of ketones increased under 400 MPa treatment conditions, while the ketone content decreased under 500 MPa treatment conditions. These findings suggest that HPP treatments of different intensities significantly affect the content of volatile flavor compounds in *M. mercenaria*.

Volatile flavor substances such as 1-penten-3-ol, pentanal, 2-butanone, 3-pentanone, (E)-2-pentenal, 2-hexenal, n-hexanol, 3-octanone, (E)-2-octenal, nonanal, (Z)-4-heptenal, 1-penten-3-one, hexanal, heptanal, benzaldehyde, and octanal were reduced after HPP treatment. As the HPP treatment pressure increased, the levels of 2-methylbutanal, ethanol, and acetone were higher in the 400 MPa group, while the content of pentanal was higher in the 500 MPa group. Aldehydes are mainly produced by the oxidation of unsaturated fatty acids or degradation of amino acids [23]. They generally have fruit, grass, fat, and fishy odor characteristics, and their odor threshold is generally low. After 400 MPa HPP treatment, increasing levels of 2-Methylbutyraldehyde with coffee and nutty flavors added pleasant flavor to *M. mercenaria*. Alcohols usually have a soft aroma, are vegetal, and have rancidity and an earthy odor. However, due to the high threshold of odor, it generally contributes little to the odor of clams [43]. Furthermore, ketones are an important product of fat oxidation; their flavor is typically described as a creamy flavor and a cheese flavor. These substances contribute significantly to the development of volatile odors in aquatic products. After HPP treatment at 400 MPa, ketones basically exhibit a decreasing trend. The decline could be because the ketone, as a carbonyl molecule, combines with amino acids, peptides, proteins, and other substances, causing its content to decrease [44]. It has been reported that hexanal, heptanal, and nonanal in aquatic products have a fishy taste [45]. In this study, HPP treatment significantly reduced the content of low-molecular-weight aldehydes and had the effect of reducing the fishy taste and improving the flavor of the clams. The study showed that HPP treatment could reduce the bitterness value of *M. mercenaria* and improve olfactory indexes such as sweetness. Moreover, the 400 MPa treatment group had better quality in olfactory indexes such as richness and sweetness.

### 2.10. Correlation Analysis between Physicochemical Parameters

**Figure 8:**
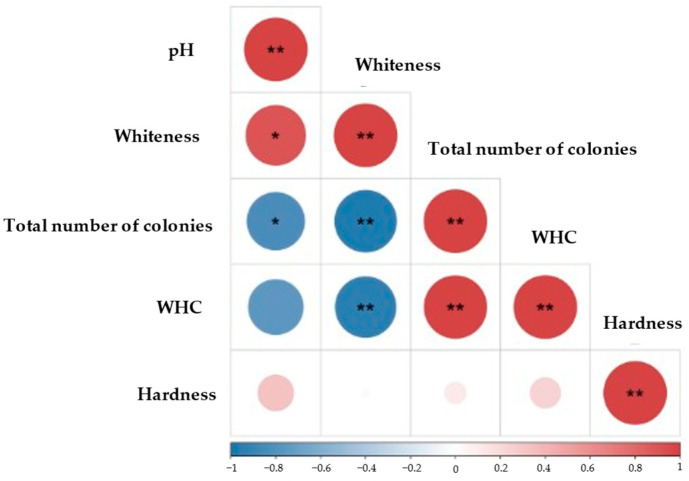
Correlation analysis of physicochemical factors of *M. mercenaria*. * and ** indicate significant differences at *p* < 0.05 and *p* < 0.01, respectively.

Pearson’s correlation analysis was carried out on the physicochemical indices (whiteness, WHC, total number of colonies, pH, and hardness) of *M. mercenaria* treated with HPP (Figure 8). Whiteness displayed a significantly negative correlation with the total number of colonies and WHC (*p* < 0.01) during different HPP treatments, while the pH was significantly negatively correlated with the total number of colonies (*p* < 0.05). A significantly positive correlation was observed between WHC and the total number of colonies (*p* < 0.01), consistent with the findings of the previous study. Furthermore, the observed correlation between whiteness and pH revealed a significantly positive relationship (*p* < 0.05). This association can be explained by the impact of muscle pH on the water-binding properties of proteins, consequently affecting the structural composition of the meat and its light-reflective properties [46].

## 3. Materials and Methods

### 3.1. Sample Preparation and HPP Treatment

*M. mercenaria* was obtained from the Tianjin Key Laboratory of Aquatic Ecology and Aquaculture Test Base, and *M. mercenaria* was loaded with crushed ice in a foam box and returned to the laboratory within 12 h. After sorting, *M. mercenaria* plants with weak viability and that were already dead were removed, and the remaining samples were mixed, placed in the laboratory and stored in sand-filtered seawater at a temperature of 24.0~25.8 °C, pH of 8.0~8.2, dissolved oxygen content of 5.87~6.88 mg/L, and salinity of 23‰ for one week to obtain uniform samples.

The sand and other debris on *M. mercenaria* were cleaned with a brush, ultrasonically cleaned for 2 min, dried with filter paper, and put into PET/PE/PA composite bags. Then, 50 mL of distilled water was added to each bag, heat sealed, and stored at 2 °C [12,47].

The packaged *M. mercenaria* underwent random division into 6 groups for processing using HPP equipment (HPPL1-600/5, Tianjin Huatai-senmiao Bioengineering Technology Co., Ltd., Tianjin, China). *M. mercenaria* was exposed to pressures of 100 MPa, 200 MPa, 300 MPa, 400 MPa, and 500 MPa for 8 min at 20 °C. A control group without treatment was included. After treatment, all pressure-treated and control clams were shucked, the meat was removed directly and packed into sealed bags, and the samples were shielded with crushed ice to minimize temperature variations and were stored at 5 °C until evaluating physical, chemical, and sensory quality aspects.

### 3.2. Determination of Nutritional Components

The ash content was determined by the magnesium acetate method. The samples were incinerated in a muffle furnace at 550 °C for 24 h [48]. The protein content was determined using the Kjeldahl nitrogen determination method, employing a conversion factor of 6.25 for nitrogen to protein [49]. The fat content was determined using the Soxhlet extraction technique. The samples were extracted with petroleum ether at 85 °C for 8 h [50].

### 3.3. Determination of pH

To determine the pH, 10 g of ground *M. mercenaria* sample was weighed and diluted with distilled water to 1:10 in the form of a suspended liquid. An MTI patting aseptic homogenizer was used to oscillate at 600 r/min for 1 min, the mixture was allowed to stand for 30 min, and the pH was measured with a pH meter [18,51].

### 3.4. Color Measurement

The L* (lightness), a* (redness), and b* (yellowness) values of the ventral body (belly section) of *M. mercenaria* meat samples were measured with chromameter (HP-2132, Hewlett-Packard, Shanghai, China). Each group contained three samples, which were each measured three times, and the average value was taken. Measurement conditions: D65 illuminant and the observer at 10°. In addition, before each series of measurements, the instrument was calibrated using a Whiteboard. The value of L represents the brightness of the sample, and a* and b* represent the color intensity from green to red and from yellow to blue, respectively due to the *M. mercenaria* being treated with HPP to make the meat creamy white. The advantage of using whiteness is better resolution of the relatively minor difference between white and yellowish in order to more directly observe the change in color of *M. mercenaria* after HPP treatment [51,52].

The whiteness value is calculated using the following Formula (1):

(1) $$\begin{array}{c} W=100-\sqrt{\left[{\left(100-L^{*}\right)}^{2}+a^{2}+b^{2}\right]} \end{array}$$

### 3.5. Total Number of Bacterial Colonies

The sample (10 g) was weighed and positioned on a clean bench. Then, 90 mL of sterile saline was introduced to the samples, followed by homogenization for 1 to 2 min. After homogenization, a 10-fold serial dilution was carried out. Subsequently, 1 mL of the sample homogenate solution was transferred into a sterile Petri dish. To this dish, 15 to 20 mL of PCA (Plate Count Agar) was added and mixed thoroughly. The Petri dishes were then placed in an incubator at a temperature of 37 ± 1 °C for a duration of 48 h. Colonies ranging from 30 to 300 were tallied on each plate, and the total number of colonies was computed and expressed as colony-forming units per gram (CFU/g).

### 3.6. Determination of Water Holding Capacity

The sample (3 g) was meticulously taken, precisely weighed, and then enveloped in filter paper before insertion into a centrifuge tube. The sample underwent centrifugation at a speed of 2000 r/min for 15 min. Subsequently, the sample was re-weighed accurately post-centrifugation. The moisture content was evaluated using the direct drying method. Each sample underwent five replicates, and the average value was computed as the final test result. The average moisture content was expressed as a% WHC.

The hydraulic retention formula is as follows:

(2) $$\begin{array}{c} WHC=\frac{W_{0}-W_{1}}{W_{0}}\times 100\% \end{array}$$

where W_0_ is the weight of the sample before centrifugation and W_1_ is the weight of the sample after centrifugation.

### 3.7. Texture Determination

The abdominal tissues and closed-shell muscles of *M. mercenaria* were analyzed using a texture analyzer (TA-XT2i, Stable Micro Systems, Godalming, UK) equipped with a P/50 flat-bottom cylindrical probe with a diameter of 50 mm. The following parameters were configured: the probe drop, test, and return speed were set to 1 mm/s; the test duration was maintained at 100 s; the trigger point load was adjusted to 5 g; and the stress relaxation compressive deformation was set at 30%. Data collection occurred at a rate of 500 Hz. Each group underwent three measurements, and the average value of the test outcomes was regarded as the final result. Hardness, cohesion, elastic adhesion, and chewability were derived from the texture profile analysis (TPA) curve [53,54].

### 3.8. Scanning Electron Microscopy (SEM)

The *M. mercenaria* meat was sliced into pieces measuring 0.5 × 0.5 × 0.5 mm. After placing a drop of cooling gel on the sample stage of the benchtop scanning electron microscope, the cut sample was placed on the sample stage with the following parameter settings: the sample stage temperature was −15 °C, the accelerating voltage was 10 kV. Subsequently, the surface microstructure of the sample was examined and photographed using SEM at a magnification of 2000 times [55,56].

### 3.9. Sensory Assessment

In the sensory evaluation, the odor score was conducted following the guidelines outlined in the method described by Jiagen Li and Wenhui Zhu et al. [57,58]. All panelists (5 males and 5 females, with a mean age of 24), all of whom had no seafood allergies, had received relevant training to ensure that they could consistently describe the attributes. Samples of the same weight of *M. mercenaria* treated with various levels of HPP were placed in labeled tasting cups and were randomly coded. The coded samples for sensory evaluation were provided to the assessment panelists in a well-lit sensory room that did not interfere with color vision, and they rinsed their mouths with water before tasting each sample. The sensory assessment results were recorded using a 10-point scale (Table S1): degree of clam meat peeling (1 = not separated, 10 = completely separated), color (1 = yellow, 10 = creamy white), odor (1 = rancid taste, 10 = fresh taste), texture (1 = loose and inelastic, 10 = tight and elastic), and overall acceptability (1 = unacceptable, 10 = acceptable).

### 3.10. GC-IMS Measurements

A gas chromatography/ion mobility spectrometry (GC-IMS) device (FlavourSpec^®^, G.A.S., Dortmund, Germany) was used in this study to assess VOCs in *M. mercenaria* meat. The analytical conditions were set up using the previously published analytical conditions by Luo et al. [59]. A 5.0 g sample was placed in a headspace vial and incubated at 50 °C at 500 r/min for 20 min. A volume of 0.5 mL was then extracted from the headspace and autosampled using a syringe at 85 °C. The samples were put into an MXT-5 column (15 m × 0.53 mm × 1 μm, Restek, Beijing, China) and eluted at 60 °C using nitrogen (99.999% purity). The carrier gas flow rate was determined using the prior study of Mi et al. [60].

The carrier gas flow rate program was as follows: 0–2 min, 2 mL/min; 2–10 min, 15 mL/min; and 10–20 min, 100 mL/min. The isolates were ionized in an IMS ionization chamber set to 45 °C and 500 V/cm voltage. The volatile components were identified using a combination of drift time (DT) and retention index (RI). The samples were qualitatively examined by evaluating n-ketone (C_4_–C_9_) calibration solutions, creating standard curves of retention index vs. retention duration, and comparing the volatiles’ DT and RI values to the built-in NIST and IMS databases. All measurements were made in five or three replications (n = 3).

### 3.11. Data Processing

Waller–Duncan difference significance analysis was conducted using SPSS 21.0 software. Each sample underwent three repeated samplings for determination, and the results were presented as mean standard deviation. Graphs were generated using Origin 9.0 software, while correlation analysis heat maps were drawn using OriginPro 2021.

## 4. Conclusions

The results revealed a significant bactericidal effect of the HPP treatment. At a pressure of 500 MPa, fundamental sterility was attained. The HPP treatment had minimal impact on the essential nutrients found in *M. mercenaria*, thereby maintaining their nutritional value. However, various attributes such as whiteness, pH, hardness, elasticity, chewability, cohesiveness, resilience, and water holding capacity experienced notable alterations. Moreover, the sensory evaluation highlighted intensified odors and discernible shifts in volatile flavor compounds. Specifically, fishy notes decreased, while flavor elements like sweetness increased noticeably. These findings suggest that the HPP treatment enhanced the taste, texture, and flavor of *M. mercenaria*, meeting consumer sensory preferences. In summary, the HPP treatment positively influenced the quality of *M. mercenaria* meat, maintaining the original nutrition and significantly enhancing its palatability, which facilitated the diversified processing of the product. These results offer valuable theoretical insights for subsequent industrial production.

## Figures and Tables

**Table 1:** Determination of basic components of *M. mercenaria* after HPP treatment.

Group/MPa	Protein (%)	Fat (%)	Ash (%)
0	66.92 ± 0.04 ^a^	7.21 ± 0.01 ^a^	21.32 ± 0.12 ^a^
100	63.62 ± 0.21 ^b^	6.15 ± 0.52 ^a^	18.35 ± 0.55 ^a^
200	63.35 ± 0.18 ^b^	6.08 ± 0.23 ^a^	17.22 ± 0.69 ^ab^
300	62.28 ± 0.35 ^bc^	5.99 ± 0.52 ^a^	16.96 ± 0.25 ^ab^
400	61.78 ± 0.25 ^c^	5.93 ± 0.68 ^a^	15.93 ± 0.36 ^c^
500	60.80 ± 0.19 ^c^	5.77 ± 0.28 ^a^	16.31 ± 0.51 ^bc^

Different letters indicate significant differences at *p* < 0.05, and the same letter indicates a non-significant difference at *p* > 0.05.

**Table 2:** Effect of different pressures on sensory quality of *M. mercenaria*.

Project	0 MPa	100 MPa	200 MPa	300 MPa	400 MPa	500 MPa
Degree of clam stripping	1.2	1.5	4.7	7.8	9.2	9.5
Scent	6.8	6.8	7.1	7.7	8.1	8.2
Texture	4.1	4.2	5.3	7.5	8.2	9.1
Color	3.7	3.9	4.2	5.5	7.1	7.5
Acceptability	3.5	3.4	4.2	7.1	8.5	7.8

**Table 3:** List of qualitative compounds in GC-IMS spectra of *M. mercenaria* treated under different pressures.

No.	Compound	CAS#	Formula	RI	Rt	Dt	Peak Intensities		
							0 MPa	400 MPa	500 MPa
	Aldehydes (19 compounds)								
1	Hexanal monomer	C66251	C_6_H_12_O	795.7	262.855	1.263	2829.77 ± 75.78 ^a^	1443.11 ± 59.45 ^c^	2263.61 ± 76.01 ^b^
2	Hexanal dimer	C66251	C_6_H_12_O	794.8	262.027	1.556	3267.56 ± 318.9 ^a^	510.5 ± 15.12 ^c^	1737.01 ± 184.49 ^b^
3	(E)-2-Pentenal monomer	C1576870	C_5_H_8_O	758.5	227.673	1.110	1325.9 ± 92.8 ^a^	453.58 ± 15.06 ^b^	485.23 ± 50.57 ^b^
4	(E)-2-Pentenal dimer	C1576870	C_5_H_8_O	756.2	225.604	1.361	786.3 ± 106.01 ^a^	72.85 ± 9.47 ^b^	78.27 ± 14.62 ^b^
5	Pentanal monomer	C110623	C_5_H_10_O	702.5	182.558	1.197	598.62 ± 36.63 ^b^	535.55 ± 43.17 ^b^	1036.03 ± 69.11 ^a^
6	Pentanal dimer	C110623	C_5_H_10_O	700.2	180.902	1.41968	196.86 ± 25.4 ^b^	71.51 ± 3.68 ^c^	304.22 ± 51.82 ^a^
7	2-Methylbutanal	C96173	C_5_H_10_O	675.4	166.416	1.18333	654.83 ± 63.37 ^a^	514.49 ± 51.42 ^b^	353.87 ± 17.79 ^c^
8	3-Methylbutanal monomer	C590863	C_5_H_10_O	658.5	158.551	1.18465	457.34 ± 42.36 ^a^	454.11 ± 51.96 ^a^	216.54 ± 10.96 ^b^
9	3-Methylbutanal dimer	C590863	C_5_H_10_O	663.9	161.035	1.39711	203.53 ± 34.18 ^a^	109.48 ± 7.97 ^b^	38.18 ± 0.9 ^c^
10	2-Hexenal monomer	C505577	C_6_ H_10_O	852.5	324.113	1.18067	673.7 ± 72.06 ^a^	185.38 ± 9.32 ^b^	226.69 ± 38 ^b^
11	2-Hexenal dimer	C505577	C_6_H_10_O	850.4	321.629	1.51794	140.59 ± 20.13 ^a^	26.8 ± 4.66 ^b^	25.44 ± 2.11 ^b^
12	Heptanal monomer	C111717	C_7_H_14_O	901.6	388.886	1.3365	2653.43 ± 129.2 ^a^	682.32 ± 119.72 ^c^	1310.56 ± 73.77 ^b^
13	Heptanal dimer	C111717	C_7_H_14_O	900.4	387.002	1.68235	1086.65 ± 95.1 ^a^	152.73 ± 8.88 ^c^	358.45 ± 46.57 ^b^
14	Benzaldehyde monomer	C100527	C_7_H_6_O	979.2	522.015	1.14755	8895.87 ± 312.3 ^a^	1943.08 ± 445.8 ^c^	5389.91 ± 1182.24 ^b^
15	Benzaldehyde dimer	C100527	C_7_H_6_O	977.1	517.857	1.47073	4758.26 ± 962.4 ^a^	507.23 ± 31 ^b^	2381.41 ± 942.55 ^c^
16	Octanal	C124130	C_8_H_16_O	1012.9	585.782	1.40354	1305.01 ± 117.9 ^a^	457.65 ± 59.24 ^c^	855.02 ± 38.78 ^b^
17	(E)-2-Octenal	C2548870	C_8_H_14_O	1068.5	693.483	1.33079	360.62 ± 17.42 ^a^	96.62 ± 32.4 ^b^	86.94 ± 3.28 ^b^
18	n-Nonanal	C124196	C_9_H1_8_O	1108.4	782.859	1.47075	245.8 ± 14.82 ^a^	94.19 ± 12.91 ^b^	99.68 ± 12.36 ^b^
19	(Z)-4-Heptenal	C6728310	C_7_H_12_O	896.6	381.569	1.15204	277.27 ± 39.2 ^a^	160.94 ± 45.82 ^ab^	190.25 ± 2.5 ^b^
Alcohols (8 compounds)									
20	1-Pentanol monomer	C71410	C_5_H_12_O	774.2	242.16	1.25636	495.08 ± 16.29 ^a^	226.06 ± 32.32 ^b^	274.45 ± 14.86 ^b^
21	1-Pentanol dimer	C71410	C_5_H_12_O	771.1	239.262	1.50997	98.37 ± 11.41 ^a^	32.22 ± 5.87 ^b^	40.03 ± 2.57 ^b^
22	1-Penten-3-ol monomer	C616251	C_5_H_10_O	700.8	181.316	0.94299	1936.14 ± 120.6 ^a^	1008.69 ± 52.31 ^b^	956.3 ± 14.57 ^b^
23	1-Penten-3-ol dimer	C616251	C_5_H_10_O	694.9	177.177	1.34931	873.54 ± 87.6 ^a^	148.29 ± 7.87 ^b^	214.01 ± 21.02 ^b^
24	Ethanol	C64175	C_2_H_6_O	502.4	103.088	1.04391	2546.55 ± 258.7 ^b^	4233.28 ± 315.1 ^a^	3837.35 ± 103.2 ^a^
25	n-Hexanol monomer	C111273	C_6_H_14_O	878.6	356.86	1.32849	1294.29 ± 310.5 ^a^	534.98 ± 421.7 ^b^	287.65 ± 21.86 ^b^
26	n-Hexanol dimer	C111273	C_6_H_14_O	870.9	346.812	1.63912	268.24 ± 64.4 ^a^	112.61 ± 64.37 ^b^	81.36 ± 10.09 ^b^
27	1-Propanol	C71238	C_3_H_8_O	577.7	126.069	1.11088	2417.2 ± 215.43 ^a^	892.97 ± 56.63 ^b^	858.62 ± 74.61 ^b^
	Ketones (7 compounds)								
28	Acetone	C67641	C_3_H_6_O	515.7	106.813	1.12756	558.58 ± 82.82 ^b^	706.97 ± 155.44 ^a^	731.95 ± 57.53 ^a^
29	2-Butanone	C78933	C_4_H_8_O	590.4	130.455	1.24698	449.45 ± 56.99 ^a^	112.3 ± 10.31 ^b^	115.75 ± 3.17 ^b^
30	1-Penten-3-one monomer	C1629589	C_5_H_8_O	691.2	174.631	1.0794	470.65 ± 34.58 ^a^	121.69 ± 6.23 ^b^	169.28 ± 15.46 ^b^
31	1-Penten-3-one dimer	C1629589	C_5_H_8_O	695.3	177.451	1.309	357.8 ± 13.95 ^a^	126.79 ± 6.79 ^b^	149.1 ± 12.81 ^b^
32	3-Pentanone	C96220	C_5_H_10_O	703.2	183.091	1.10996	225.39 ± 7.18 ^a^	144.18 ± 4.83 ^b^	144.33 ± 3.92 ^b^
33	3-Octanone monomer	C106683	C_8_H_16_O	994	552.237	1.30246	552.86 ± 21.25 ^a^	129.02 ± 17.83 ^c^	186.62 ± 24.21 ^b^
34	3-Octanone dimer	C106683	C_8_H_16_O	989.8	543.459	1.71567	286.92 ± 28.47 ^a^	65.7 ± 16.5 ^b^	79.73 ± 13.73 ^b^
	Unknown (17 compounds)								
35	Unknown-1	/	/	581.4	127.322	581.4	472.38 ± 57.08 ^a^	649.55 ± 49.9 ^a^	673.08 ± 14.99 ^a^
36	Unknown-2	/	/	664.9	161.472	664.9	285.14 ± 11.39 ^a^	116 ± 6.31 ^b^	106.25 ± 10.38 ^b^
37	Unknown-3	/	/	699.3	180.271	699.3	382.95 ± 39.6 ^a^	267.68 ± 13.67 ^b^	384.26 ± 54.5 ^a^
38	Unknown-4	/	/	730.4	203.769	730.4	215.52 ± 6.17 ^a^	97.57 ± 20.78 ^ab^	74.23 ± 5.68 ^a^
39	Unknown-5	/	/	750.2	220.374	750.2	255.18 ± 6.69 ^a^	83.83 ± 5.09 ^b^	78.34 ± 5.28 ^b^
40	Unknown-6	/	/	747	217.554	747	308.05 ± 15.68 ^a^	108.76 ± 8.64 ^b^	130.44 ± 6.6 ^b^
41	Unknown-7	/	/	749.5	219.747	749.5	228.64 ± 8.6 ^a^	19.34 ± 1.25 ^b^	18.83 ± 1.23 ^b^
42	Unknown-8	/	/	830.6	299.014	830.6	302.52 ± 2.61 ^a^	65.05 ± 7.82 ^b^	75.5 ± 1.65 ^b^
43	Unknown-9	/	/	987.1	537.873	987.1	293.96 ± 5.17 ^a^	473.1 ± 12.67 ^b^	274.47 ± 53.09 ^c^
44	Unknown-10	/	/	991.8	547.449	991.8	578.63 ± 106.42 ^a^	91.11 ± 22.82 ^b^	119.12 ± 20.22 ^b^
45	Unknown-11	/	/	11,088	783.657	1108.8	1168.29 ± 46.49 ^a^	196.01 ± 33.9 ^b^	209.29 ± 33.69 ^b^
46	Unknown-12	/	/	11,078	781.263	1107.8	507.53 ± 20.14 ^a^	86.16 ± 14.38 ^b^	79.56 ± 6.76 ^b^
47	Unknown-13	/	/	983.6	530.676	983.6	294.7 ± 5.52 ^b^	55.92 ± 8.36 ^b^	174.46 ± 38.37 ^a^
48	Unknown-14	/	/	875	352.102	875	239.97 ± 7.55 ^a^	35.51 ± 2.61 ^b^	32.56 ± 6.4 ^b^

Different letters indicate significant differences at *p* < 0.05, and the same letter indicates a non-significant difference at *p* > 0.05.

## Data Availability

Data will be made available on request.

## Supplementary Materials

The following supporting information can be downloaded at: https://www.mdpi.com/article/10.3390/molecules29184466/s1, Table S1: Standard of sensory evaluation; Figure S1: GC-IMS plots of volatile compounds of *M. mercenaria* at different pressures.
