# Supplementary material for: Effect of High-Pressure Processing Treatment on the Physicochemical Properties and Volatile Flavor of Mercenaria mercenaria Meat
Source: Molecules. 2024 Sep 20;29(18):4466. doi: 10.3390/molecules29184466 (PMC11433659; doi:10.3390/molecules29184466)
Supplement: Supplementary file 1 [file molecules-29-04466-s001.zip › molecules-3144999-supplementary.pdf]

Table S1. Standard of sensory evaluation

Project	Scoring Criteria	Score（1-10）
Degree of clam meat peeling	Not separated	1~3
	Partially separated	4~6
	Fully separated	7~10
Scent	Sour taste	1~3
	Seaweed flavor	4~6
	Fresh taste	7~10
Texture	Loose and inelastic	1~3
	Slightly elastic	4~6
	Muscle tight and elastic	7~10
Color	Yellow	1~3
	Beige	4~6
	Milk white	7~10

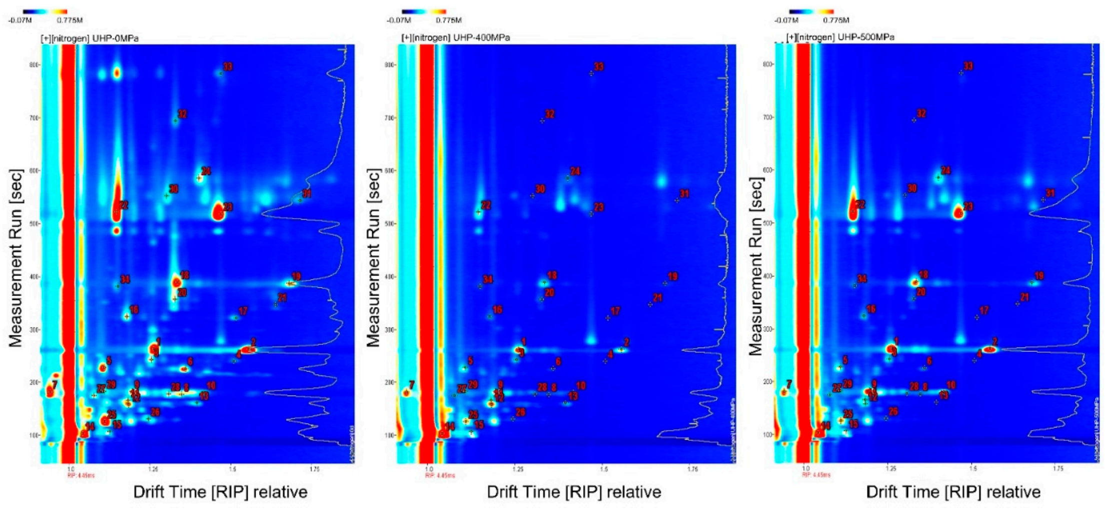

Figure S1: GC-IMS plots of volatile compounds of *M. mercenaria* at different pressures
